# Membrane transporters and protein traffic networks differentially affecting metal tolerance: a genomic phenotyping study in yeast

**DOI:** 10.1186/gb-2008-9-4-r67

**Published:** 2008-04-07

**Authors:** Roberta Ruotolo, Gessica Marchini, Simone Ottonello

**Affiliations:** 1 Department of Biochemistry and Molecular Biology, Viale G.P. Usberti 23/A, University of Parma, I-43100 Parma, Italy

## Abstract

**Background:**

The cellular mechanisms that underlie metal toxicity and detoxification are rather variegated and incompletely understood. Genomic phenotyping was used to assess the roles played by all nonessential *Saccharomyces cerevisiae *proteins in modulating cell viability after exposure to cadmium, nickel, and other metals.

**Results:**

A number of novel genes and pathways that affect multimetal as well as metal-specific tolerance were discovered. Although the vacuole emerged as a major hot spot for metal detoxification, we also identified a number of pathways that play a more general, less direct role in promoting cell survival under stress conditions (for example, mRNA decay, nucleocytoplasmic transport, and iron acquisition) as well as proteins that are more proximally related to metal damage prevention or repair. Most prominent among the latter are various nutrient transporters previously not associated with metal toxicity. A strikingly differential effect was observed for a large set of deletions, the majority of which centered on the ESCRT (endosomal sorting complexes required for transport) and retromer complexes, which - by affecting transporter downregulation and intracellular protein traffic - cause cadmium sensitivity but nickel resistance.

**Conclusion:**

The data show that a previously underestimated variety of pathways are involved in cadmium and nickel tolerance in eukaryotic cells. As revealed by comparison with five additional metals, there is a good correlation between the chemical properties and the cellular toxicity signatures of various metals. However, many conserved pathways centered on membrane transporters and protein traffic affect cell viability with a surprisingly high degree of metal specificity.

## Abstract 2

Genomic phenotyping was used to assess the role of all non-essential S. cerevisiae proteins in modulating cell viability after exposure to cadmium, nickel and other metals.

## Background

Metals, especially the nonessential ones, are a major environmental and human health hazard. The molecular bases of their toxicity as well as the mechanisms that cells have evolved to cope with them are rather variegated and incompletely understood. The soft acid cadmium and the borderline acid nickel are nonessential transition metals of great environmental concern. Although redox inactive, cadmium and nickel cause oxidative damage indirectly [1] and they both have carcinogenic effects [2,3], albeit with reportedly different mechanisms [1,4-6].

The cellular effects of cadmium are far more studied than those of nickel. Instrumental to the elucidation of some of the basic mechanisms that underlie cadmium toxicity has been the model eukaryote *Saccharomyces cerevisiae *[7]. It was studies conducted in this organism, for example, that yielded the first demonstration of the indirect nature of cadmium's genotoxic effects, which leads to genome instability by inhibiting DNA mismatch repair [8] and other DNA repair systems [6]. Similarly, lipid peroxidation as a major mechanism of cadmium toxicity [9] as well as the central roles played by thioredoxin and reduced glutathione (GSH) [7], and vacuolar transport systems such as Ycf1 [10], in cadmium detoxification were first documented in yeast. Some of the above components were shown to be upregulated at both the mRNA [11,12] and protein [12,13] levels in cadmium-stressed yeast cells. Predominant among these expression changes was the upregulation of the sulfur amino acid biosynthetic pathway and the induction of isozymes with a markedly reduced sulfur amino acid content as a way to spare sulfur for GSH synthesis [12]. A number of additional cadmium-responsive genes without any obvious relationship to sulfur sparing or cadmium stress were also identified, however. Curiously, only a small subset of the most cadmium-responsive genes produce a metal-sensitive phenotype when deleted [13], thus reinforcing the notion that transcriptional modulation *per se *is not a general predictor of the pathways influencing stress tolerance [14,15]. For example, deletion of genes coding for two major organic peroxide-scavenging enzymes (GPX3 and AHP1; the latter encoding a cadmium-induced alkyl hydroperoxide reductase) did not impair cadmium tolerance [13].

By comparison, only a few studies have dealt with nickel toxicity in yeast. Interestingly, they showed that unprogrammed gene silencing, which is a major mechanism of nickel toxicity and carcinogenicity in humans [16,17], also operates in *S. cerevisiae*. This further emphasizes the high degree of conservation of various aspects of metal toxicity as well as the usefulness of *S. cerevisiae *as a model organism for elucidating the corresponding pathways in humans. They also suggest, however, that a broad and as yet largely unexplored range of cellular pathways may be involved in alleviating the toxic effects of metals. What is currently missing, in particular, is a global view of such pathways at the phenotype level and a genome-wide comparison of different metals as well as other stressors.

We have addressed these issues by examining the fitness of a genome-wide collection of yeast deletion mutant strains [18,19] exposed to two chemically diverse metals, namely cadmium and nickel, each of which is a known carcinogen [2,3,20]. This allowed us to assess the role of all nonessential proteins in modulating the cellular toxicity (sensitivity or resistance) of these two metals. The results of this screen were integrated with interactome data and compared with the genomic phenotyping profiles of other stressors. To gain further insight into the cytotoxicity signatures of different metals, the entire set of 388 mutants exhibiting an altered viability after exposure to cadmium and nickel was challenged with four additional metals (mercury, zinc, cobalt and iron) plus the metalloid AsO_2_^-^. Although overall there is good correlation between the chemical properties and the cellular toxicity signatures of various metals, many conserved pathways centered on (but not limited to) membrane transporters and protein traffic affect cell viability with a surprisingly high degree of metal specificity.

## Results and discussion

### Genomic phenotyping of cadmium and nickel toxicity

Sublethal concentrations of 50 μmol/l cadmium and 2.5 mmol/l nickel (see 'Materials and methods', below, for details) were used for multireplicate screening of the yeast haploid deletion mutant collection (five replicates for each metal), which was performed by manually pinning ordered sets of 384 strains onto metal-containing yeast extract-peptone-dextrose (YPD)-agar plates (Additional data file 1 [Figure S1A]). After culture and colony size inspection, strains scored as metal sensitive or resistant in at least three screens were individually verified by spotting serial dilutions onto metal-containing plates. Mutant strains exhibiting various levels of metal sensitivity (high sensitivity [HS], medium sensitivity [MS], and low sensitivity [LS]) and a single class of metal resistant mutant strains were recognized (Additional data file 1 [Figures S1B and S1C]).

**Figure 1:**
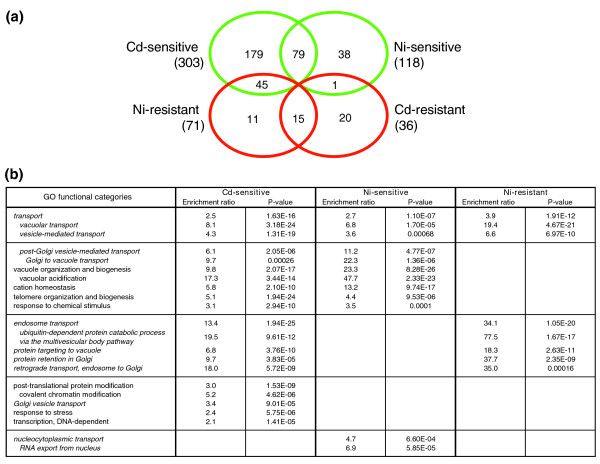
Distribution among different sensitivity/resistance groups and functional classification of metal tolerance affecting mutations. **(a) **Venn diagram visualization of mutant strains displaying multimetal or metal-specific sensitivity (green circles) or resistance (red circles); also shown are mutants characterized by an opposite phenotypic response to the two metals (45 cadmium sensitive/nickel resistant strains and one cadmium resistant/nickel sensitive strain). **(b) **Biologic processes associated with metal toxicity-modulating genes identified with the Gene Ontology (GO) Term Finder program [99]. Statistical significance of GO term/gene group association (*P*-value < 0.001) and enrichment ratios are reported for each category; parent terms are presented in bold, and child terms of the parent class 'transport' are presented in italics.

A total of 388 mutant strains that were sensitive or resistant to cadmium and/or nickel were identified. As shown in Figure 1a, some of them were specifically sensitive or resistant to cadmium or nickel, whereas others exhibited an altered tolerance to both metals. Metal-sensitive mutants exceeded the resistant ones by more than threefold. The number of sensitive mutants was considerably higher for cadmium than for nickel, which is in accordance with the strikingly different cellular toxicity previously reported for these two metal ions in animal cells [4,21]. Conversely, mutants resistant to nickel were significantly more abundant than cadmium-resistant mutant strains. More than two-thirds of the nickel-resistant mutants were found to be sensitive to cadmium, as opposed to only one instance of cadmium resistance/nickel sensitivity (*smf1Δ*). A detailed list of the mutants, including their degree of sensitivity (Additional data file 1 [Figures S1B and S1C]), Gene Ontology (GO) description, and related information, is provided in Additional data file 2. Human orthologs were identified for about 50% of the genes causing metal sensitivity or resistance, 27 of which correspond to genes previously found to be involved in human diseases, especially cancer. Twenty-four mutants are deleted in genes encoding uncharacterized open reading frames (ORFs), whereas four metal toxicity modulating genes are homologous to unannotated human ORFs (Additional data file 2). Genomic phenotyping data were also compared with the results of transcriptomic analyses conducted on cadmium-treated yeast cells [11]. In keeping with previous comparisons of this kind [14,15], only a marginal (about 7%) overlap was detected (Additional data file 2).

As revealed by the GO analysis summarized in Figure 1b, a wide range of cellular processes is engaged in the modulation of cadmium and nickel toxicity. At variance with cadmium resistant mutants, which are scattered throughout various GO categories, nickel-resistant as well as cadmium/nickel-sensitive mutant strains were found to be enriched in specific functional categories. Some of the top responsive genes identified by previous expression profiling studies (for example, genes involved in GSH and reduced sulfur metabolism [11,13]) were found to be among deletion mutants specifically sensitive to cadmium, especially within the 'response to stress' category. As expected for cells treated with agents that are actively internalized by and sequestered into vacuoles, a number of the most significant GO categories are related to 'transport', particularly to the vacuole, and to the biogenesis and functioning (for example, acidification) of this organelle. Several processes not so obviously associated with metal tolerance were also identified. For example, 'nucleocytoplasmic transport' (including nuclear pore complex formation, and functionality) emerged as a process that is specifically impaired in nickel-sensitive mutants. Other processes centered on vesicle-mediated transport also profoundly influence cadmium and nickel tolerance in different, often contrasting ways. For example, many 'Golgi-to-vacuole transport' mutants appear to be sensitive to both cadmium and nickel, whereas defects in 'endosome transport' and 'retrograde transport endosome-to-Golgi' render cells sensitive to cadmium but resistant to nickel (see below).

Importantly, mutants with metal sensitivity phenotypes of varying severity (Additional data files 1 and 2) are present within different mutant classes as well as functional categories. This discounts the possibility that only highly sensitive mutant strains or particular classes of genes are relevant to cadmium/nickel tolerance, and suggests that a suite of pathways, much broader than previously thought, modulates metal tolerance in eukaryotic cells.

### Mutations impairing cadmium and nickel tolerance

**Figure 2:**
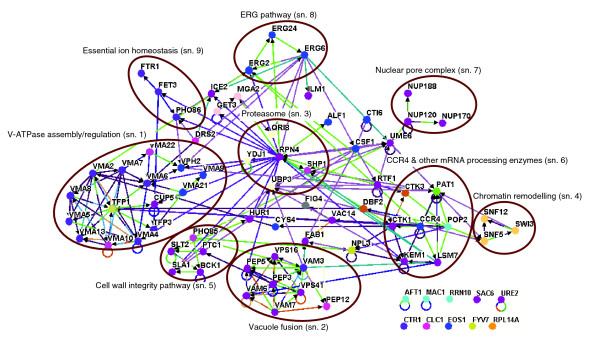
Interaction subnetworks among gene products whose disruption causes cadmium/nickel sensitivity. Physical (110) and genetic (105) interactions were identified computationally using the Network Visualization System Osprey [103]. Gene products are represented as nodes, shown as filled circles colored according to their Gene Ontology (GO) classification; interactions are represented as node-connecting edges, shown as lines, colored according to the type of experimental approach utilized to document interaction as specified in the BioGRID database [22] and in the Osprey reference manual. The nine identified subnetworks (a minimum of three interacting gene products sharing at least one GO biologic process annotation and connected by at least two physical or genetic interactions; see 'Materials and methods') are encircled and associated with a general function descriptor. Thirteen interacting gene products whose interaction or functional similarity features do not satisfy the above criterion are shown outside encircled subnetworks; genes without any reported interaction (or linked via essential genes, not addressed in this study) are shown at the bottom. Individual subnetworks were subjected to independent verification by serial dilution growth assays carried on at least two untagged strains of the opposite mating type (see 'Materials and methods'). sn., subnetwork.

To gain a more detailed understanding of metal toxicity-modulating pathways and the way in which they are interconnected, we set out to analyze genome phenotyping data in the framework of the known yeast interactome [22-24]. The 79 genes that when mutated cause sensitivity to both cadmium and nickel were initially addressed. As shown in Figure 2, 52 of these genes were identified as part of nine functional subnetworks (a minimum of three gene products sharing at least one GO biological process annotation and connected by at least two physical or genetic interactions; see 'Materials and methods', below, for details on this analysis). Seventeen of the remaining genes could be traced to a particular subnetwork but did not pass the above criterion, whereas the other ten remained as 'solitary' entries. Metal sensitivity phenotypes for at least two deletion mutants randomly sampled from each subnetwork were confirmed by independent serial dilution assays carried out on untagged strains of the opposite mating type (data not shown).

In accordance with the tight relationship between metal tolerance and vacuole functionality highlighted by GO analysis, the most populated subnetwork (subnetwork 1; *P*-value < 1.5 × 10^-18^) comprises a large set of subunits, assembly factors, and regulators of V-ATPase, which is the enzyme responsible for generating the electrochemical potential that drives the active accumulation of various ions within the vacuole [25]. Also related to V-ATPase functionality (although not included in subnetwork 1) is Cys4, which is the first enzyme of cysteine biosynthesis, whose disruption indirectly interferes with vacuolar H^+^-ATPase activity [26]. Another highly populated subnetwork (subnetwork 2; *P*-value < 2 × 10^-5^) contains eight additional vacuole-related genes belonging to either class B or C 'vacuolar protein sorting' (*vps*) mutants, whose deletion respectively causes a fragmented vacuole morphology or lack of any vacuole-like structure [27,28]. This indicates that defects in specific aspects of vacuole functionality as well as in late steps of vesicle transport to, and fusion with, the vacuole cause sensitivity to both metal ions. In keeping with this view, three additional proteins (Fab1, Fig4, and Vac14), which also cause cadmium/nickel sensitivity when disrupted, control trafficking to the vacuolar lumen [29,30]. The role played by the vacuole in metal toxicity modulation may entail both metal sequestration within this organelle as well as the clearance of metal-damaged macromolecules.

Connected with these vacuole-related hot spots, which include a number of genes previously associated with cadmium (but not nickel) tolerance [7], are five additional subnetworks. One of them (subnetwork 3; *P*-value < 7 × 10^-2^) comprises the master regulator Rpn4, which is required for proteasome biogenesis, and three ubiquitin-related proteasomal components (Qri8, Shp1, and Ubp3), thus reinforcing the notion that abnormal protein degradation plays an important role in toxic metal tolerance [31-33]. Other components previously associated with tolerance to cadmium and to other stressors include three subunits of the chromatin remodeling complex SWI/SNF (SWItch/Sucrose NonFermenting; subnetwork 4; *P*-value < 0.1) [34] and a group of regulators of the cell wall integrity/mitogen-activated protein kinase signaling pathway (subnetwork 5; *P*-value < 3.4 × 10^-6^) [35,36]. These are functionally linked to the second largest subnetwork (subnetwork 6; *P*-value < 9.1 × 10^-5^), which is centered on Ccr4 and its associated proteins. Ccr4 is a multifunctional mRNA deadenylase that can be part of mRNA decay as well as transcriptional regulatory complexes in association with the NOT factors [37]. None of the *NOT *deletion mutants was identified as metal sensitive, whereas a few other transcriptional regulators interacting with Ccr4 (for example, Dbf2 and Rtf1) cause cadmium/nickel sensitivity when disrupted. Pop2, another major deadenylase in *S. cerevisiae *[37], along with three additional RNA processing enzymes (Kem1, Lsm7, and Pat1), were also found among cadmium/nickel sensitive mutants. Previously known to be involved in the response to DNA damaging agents [38], these proteins thus appear to play a role also in metal tolerance, which might be aimed at ensuring proper translational/metabolic reprogramming under stress conditions. This finding, along with the identification of cadmium/nickel-sensitive mutations affecting three nuclear pore complex subunits (subnetwork 7; *P*-value < 7.3 × 10^-4^) and a mRNA export factor (Npl3), points to mRNA decay and trafficking (particularly nuclear export) as a novel hot spot of metal toxicity.

The last two subnetworks pertain to ergosterol biosynthesis (subnetwork 8; *P*-value < 9.8 × 10^-4^), which critically influences the structural and functional integrity of the plasma membrane (Additional data file 1 [Figure S1B] shows a representative phenotype), and to essential ion homeostasis (subnetwork 9; *P*-value < 0.12). The latter includes the endoplasmic reticulum exit protein Pho86, which is required for plasma membrane translocation of the Pho84 phosphate transporter, the high-affinity iron transport complex Ftr1/Fet3, and a transcription factor (encoded by the solitary gene *AFT1*) that positively regulates *FTR1*/*FET3 *expression. All these genes cause cadmium/nickel sensitivity when mutated. A possible explanation for this finding is that toxic metals can make iron, and other essential ions, limiting for cell growth (see below). In fact, one copper transporter (Ctr1) and a copper uptake-related transcription factor (Mac1) were also found among the cadmium/nickel-sensitive mutants in our screen.

### Metal-specific sensitive mutants

A similar interactome analysis was applied to deletion mutants that proved to be specifically sensitive to nickel or cadmium. As shown in Table 1 (and Additional data files 3 and 4), this led to the identification of seven metal-specific subnetworks and to the inclusion of nickel and cadmium specific mutants into previously identified subnetworks. Especially noteworthy are the nickel-specific expansion of the nuclear pore complex (subnetwork 7; *P*-value < 1 × 10^-4^) and the many cadmium-specific mutants added to subnetwork 4 (*P*-value < 1.7 × 10^-3^), which includes various components of the chromatin modification complexes SAGA and INO80, plus the histone deacetylase HDA1. Proteins involved in histone acetylation may affect metal tolerance by influencing DNA reactivity as well as DNA accessibility to repair enzymes, or by influencing the expression of genes needed for recovery. The selective enrichment of cadmium-sensitive mutants within this subnetwork (as well as in the cadmium-specific subnetwork 'DNA repair'; subnetwork 12; see below) is not too surprising, if one considers the known genotoxic effects of cadmium, caused by interference with DNA repair [6,8].

**Table 1:** Subnetwork organization of gene products whose disruption specifically affects nickel or cadmium tolerance

Subnetworks^a^	Nickel		Cadmium	
	Interacting gene products	Functionally linked gene products^b^	Interacting gene products^3^	Functionally linked gene products^b,c^

V-ATPase assembly/regulation (sn. 1)	Rav1, Vma16, Vph1			
Proteasome (sn. 3)	Cue1		Bre5, Cdc26, Doa1, Hlj1, Sel1, Ubi4, Ubp6, Ump1	Dia2
Chromatin assembly/remodelling (sn. 4)			SAGA complex (Ada2, Chd1, Gcn5, Hfi1, Ngg1, Spt7*, Spt20); Ino80 complex (Arp5, Arp8, Taf14); COMPASS complex (Bre2, Sdc1); Asf1, Ard1, Eaf7*, Esc2, Hda1*, Hmo1, Ioc2	Hmo1
Cell wall integrity pathway (sn. 5)		Whi3	Bem2, Dom34, Ecm33, Kcs1, Pin4, Pog1, Rvs161, Rvs167, Sic1, Sit4*, Sur7, Swi4, Swi6, Whi2	
CCR4 and other mRNA processing enzymes (sn. 6)			Dhh1	Paf1
Nuclear pore complex (sn. 7)	Nup84, Sac3, Thp1			
Essential ion homeostasis (sn. 9)	Pho88	Ccc2, Zap1	Smf3	Gef1, Pho89
AP-3 complex (sn. 10)	Apl5, Apl6, Apm3, Aps3			
General transcription (sn. 11)	Mft1, Rpb9, Rtt103, Thp2		Mediator complexes (Gal11, Med2, Pgd1, Spt21, Srb8*, Srb10); Cad1, Elp4, Tup1, Yap1	Mss11
DNA repair (sn. 12)			Ctf4, Him1, Met18, Mms22, Mre11, Pol32, Rad6, Rad27, Xrs2	
Antioxidant defense (sn. 13)			Atx2, Ccs1, Sod1, Sod2	Cad1, Glr1, Gsh1, Gsh2, Yap1, Zwf1
Hog1 pathway (sn. 14)			Fps1, Hog1, Pbs2, Rck2, Ste11	Gre2
Vesicle targeting to, from or within Golgi (sn. 15)			Erv41, Erv46, Get2, Sac1, Sec22, Sec66; Vps13; Cog5, Cog8; Pep7, Tlg2, Vps3, Vps9, Vps21, Vps45; Arl1, Arl3, Ent3, Gga2, Nhx1*, Rgp1, Ric1, Sys1, Yil039w*, Vps51, Vps54, Ypt6; Vam10*, Vps1*, Vps8*; Pep8*, Vps5*, Vps17*, Vps29*, Vps30*, Vps35*, Vps38*	Apm2, Snx3*
Ubiquitin-dependent sorting to the multivesicular body pathway (sn. 16)			Vps27*; ESCRT I complex (Vps28*, Mvb12*, Srn2*, Stp22*); ESCRT II complex (Snf8*, Vps25*, Vps36*); ESCRT-III complex (Did4*, Snf7*, Vps20*, Vps24*); Bro1*, Did2*, Doa4*, Vps4*	Bsd2*, Bul1*, Nhx1*, Tre1*

^a^Subnetworks 1 to 9 are the same as those described in Figure 2 but include deletion mutants specifically sensitive to nickel or cadmium (no nickel or cadmium specific mutants were identified for subnetworks 2 and 8); subnetworks 10 to 16 are newly identified interaction networks comprised of gene products causing nickel-specific or cadmium-specific sensitivity when disrupted (also see Additional data files 3 and 4). ^b^Gene products for which no physical or genetic interaction is documented in the BioGRID database [22] but for which a functional relationship with the indicated subnetworks has been reported. ^c^Gene mutations causing cadmium sensitivity but nickel resistance are marked with an asterisk. AP-3, Adaptor Protein-3; CCR, Carbon Catabolite Repression; ESCRT, endosomal sorting complexes required for transport; sn., subnetwork.

Only one of the new subnetworks (subnetwork 10; *P*-value < 1.6 × 10^-3^) was found to be specifically associated with nickel sensitivity (Table 1 and Additional data file 3). This includes various components of a multiprotein complex (Adaptor Protein complex AP-3) that is involved in the alkaline phosphatase (ALP) pathway for protein transport from the Golgi to the vacuole. At variance with the other Golgi-to-vacuole transport route (the so-called 'carboxypeptidase Y' [CPY] pathway), which proceeds through an endosome intermediate and includes a number of components that when disrupted cause cadmium sensitivity (see subnetwork 15 in Table 1), the ALP pathway directly targets its cargo proteins to the vacuole. Different metals and/or different metal-specific detoxifying proteins thus appear to be differentially trafficked through the Golgi-vacuole network. A similar differential toxicity effect was recently reported for iron and copper [39]. Also notable in this regard is the observation that mutants impaired in the retrieval of receptors from the endosome to the Golgi (subnetwork 15; *P*-value < 2.4 × 10^-3^) and in endosome-to-vacuole transport (subnetwork 16; *P*-value < 1.6 × 10^-8^) are specifically sensitive to cadmium but resistant to nickel (see below).

The other cadmium-specific subnetworks are 'DNA repair' (subnetwork 12; *P*-value < 0.16), which includes the ubiquitin-conjugating DNA repair enzyme RAD6; 'antioxidant defence' (subnetwork 13; *P*-value < 5.8 × 10^-2^) and other functionally related components (Table 1 and Additional data file 4); and the Hog1 kinase cascade (subnetwork 14; *P*-value < 3.7 × 10^-2^), which was previously shown to be involved in cadmium tolerance [40]. The latter, along with the upstream-acting kinase Pbs2, controls a number of cell wall integrity-related genes. Other genes that when mutated cause cadmium or nickel sensitivity encode plasma membrane (Mal31 and Smf1) and intracellular (Ccc2, Pho88, Pho89, Smf3, Ybt1, and Ycf1) transporters (or transport-related proteins), for most of which involvement in toxic metal mobilization (especially export or reduced uptake) has not previously been reported (see below).

A previously underestimated variety of cellular processes, operating in different subcellular compartments (vacuole, Golgi, and endosome, but also cytosol, nucleus, and plasma membrane), thus appears to be involved in metal tolerance in yeast. Perhaps the most significant among the novel metal toxicity-related processes revealed by our screen are mRNA decay and nucleocytoplasmic transport, and the different protein trafficking (particularly vacuole-to-Golgi) pathways that differentially affect cadmium and/or nickel tolerance when disrupted.

### Cadmium and nickel interfere with iron homeostasis through different mechanisms

**Figure 3:**
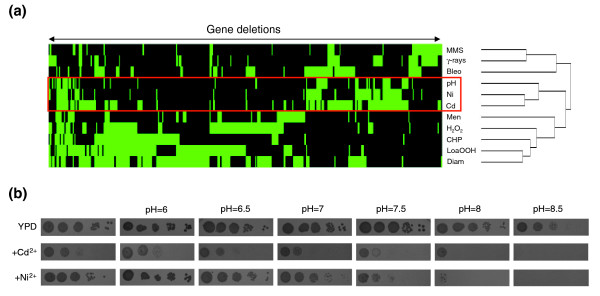
Cross-comparison with other stressors. **(a) **Hierarchical clustering of cadmium and/or nickel sensitivity-conferring mutations with the mutant sensitivity profiles of other stressors [41-45]. The x-axis corresponds to gene deletions and the y-axis indicates the various stressors; mutant strains exhibiting either an enhanced sensitivity or no phenotype are shown in green and black, respectively. Nonmetal stressors were selected from previous genomic phenotyping screens conducted on the deletion mutant collections: methyl methane sulfonate (MMS), γ-radiation (γ-rays), bleomycin (Bleo), alkaline pH (pH), menadione (Men), hydrogen peroxide (H_2_O_2_), cumene hydroperoxide (CHP), linoleic acid 13-hydroperoxide (LoaOOH), and diamide (Diam). Mutant strains were hierarchically clustered with EPCLUST (average linkage, uncentered correlation [104]); only mutants sensitive to at least two different stressors were taken into account for this analysis. **(b) **Serial dilution assays (tenfold increments from left to right, starting from an optical density at 600 nm [OD_600_] of 1.0) of wild-type cells grown in the absence (upper row) or in the presence of cadmium or nickel, on either standard yeast extract-peptone-dextrose (YPD) medium or on the same medium buffered at the indicated pH values (see 'Materials and methods' for details).

To highlight potential commonalities between cadmium/nickel exposure and other stresses, we compared our data with those obtained from similar genomic phenotyping studies [41-45]. As shown in Figure 3a, alkaline pH exhibited the closest overlap with cadmium/nickel stress. About 50% of the cadmium/nickel co-sensitive mutants (plus additional metal-specific mutants) correspond to genes previously shown to cause alkaline pH sensitivity when disrupted [44]. Furthermore, the toxicity phenotypes of both metals (particularly nickel) were exacerbated by increasing growth medium pH (Figure 3b). Especially notable among these shared (toxic metal/alkaline pH sensitive) mutants are those deleted in components directly or indirectly involved in iron homeostasis (for example, Aft1, Ctr1, Fet3/Ftr1, and Mac1), disruption of which leads to iron deficiency [46]. The latter has been implicated as a major determinant of alkaline pH stress through a reduction of iron solubility/availability [44] as well as a contributing factor to the stress induced by zinc overload in yeast, which has been shown to be caused by competition between zinc and iron at the level of cellular uptake [47]. Moreover, exposure to cadmium and nickel reduces intracellular iron levels in plant and animal cells [48-51]. We thus addressed the relationship between iron deficiency and cadmium/nickel toxicity by testing the effect of increasing iron concentrations on the fitness of cells lacking either subunit of Fet3/Ftr1 (deletion of which causes a genetic surrogate of iron starvation) exposed to either cadmium or nickel.

**Figure 4:**
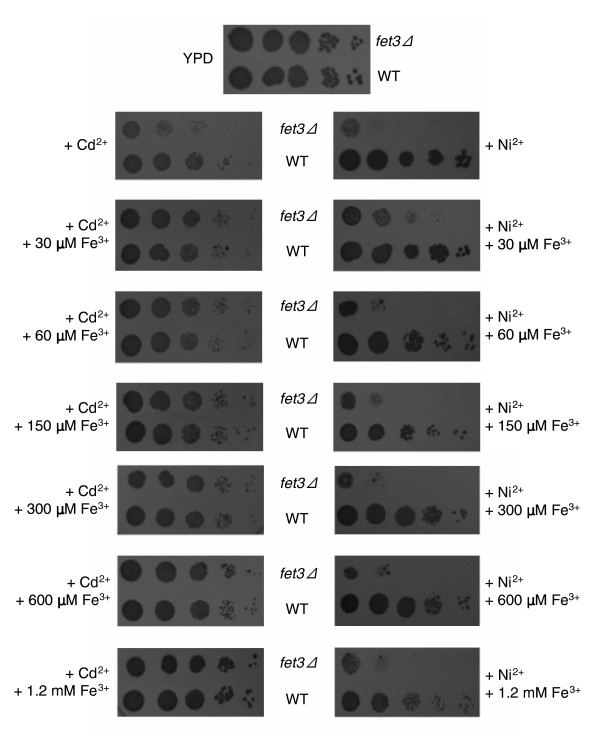
Effect of iron supplementation on cadmium and nickel tolerance. Serial dilution assays comparing the iron uptake impaired deletion mutant strain *fet3Δ *and wild-type (WT) cells grown in the presence of cadmium (40 μmol/l) or nickel (2.5 mmol/l) and supplemented with the indicated concentrations of FeCl_3_. A no-metal control is shown at the top; similar results (not shown) were obtained with a strain deleted in *FTR1*, the other component of the Fet3/Ftr1 high-affinity iron uptake system. YPD, yeast extract-peptone-dextrose.

As shown in Figure 4, supplementation of 30 μmol/l FeCl_3 _increased cadmium/nickel tolerance in *fet3Δ *cells (same results for the *ftr1Δ *mutant; data not shown). An ameliorating effect of iron supplementation was observed with other mutants not so closely related to iron homeostasis (for example, *erg2Δ*, *slt2Δ*, *vam7Δ*, and *vps51Δ*; data not shown), suggesting that iron deficiency is indeed an important (albeit indirect) determinant of cadmium/nickel toxicity. However, it should be noted that - at variance with cadmium, whose toxicity was progressively alleviated by increasing iron concentrations even in wild-type (WT) cells - nickel toxicity was only partly relieved in the *fet3Δ *mutant within a narrow, 30 to 60 μmol/l FeCl_3 _supplementation range, and gradually deteriorated thereafter (Figure 4).

Also apparent in Figure 4 is the different degree of cadmium/nickel sensitivity of the *fet3Δ *mutant (same for *ftr1Δ*), which is only moderately sensitive to cadmium (LS phenotype) but highly sensitive to nickel (HS phenotype). Other distinguishing features of the iron-related phenotypes of cadmium and nickel originate from the low-affinity/low-specificity transporters encoded by the *FET4 *and *SMF1 *genes [46,52]. These transporters become major entry sites for iron under iron overload or *fet3*/*ftr1Δ *conditions [53,54] as well in the absence of the transcription factor Aft1, which positively regulates *FET3 *and *FTR1*, whose deletion causes a HS phenotype for both cadmium and nickel (Additional data file 1 [Figure S1B] shows a representative phenotype). In addition to iron, Fet4 and Smf1 internalize other metals such as manganese, copper and cadmium [52,55,56], whereas no conclusive data on nickel have thus far been reported. In keeping with this notion, we find that *fet4 *and *smf1 *deletion mutants are cadmium (but not nickel) resistant, whereas disruption of Rox1 - a negative regulator of *FET4 *- makes cells selectively sensitive to cadmium (Additional data file 5). Conversely, over-expression of Smf1 causes cadmium (but not nickel) sensitivity (see Figures 7 and 8, below, for representative phenotypes). Therefore, even though cadmium and nickel toxicity is exacerbated at alkaline pH and both interfere with iron homeostasis, they probably do so with different mechanisms.

Cadmium, but not nickel, is internalized by broad-range transporters such as Fet4, which accumulate under iron-limiting conditions as a way to cope with iron deficiency [54]. Two nonmutually exclusive mechanisms may thus explain the alleviating effect of iron supplementation on cadmium toxicity, in both WT and *fet3Δ *cells: competition between the two metals at the level of cellular uptake; and downregulation of promiscuous (iron/cadmium) transporters under conditions of iron overload [54,57]. Competitition between iron and cadmium at the level of cellular uptake may account, for instance, for the anti-cadmium effect of iron that has been described in rats fed with a iron-supplemented diet [58]. Nickel, instead, interferes with iron homeostasis via an as yet unidentified mechanism, which does not appear to rely on direct competition with iron at the level of cellular uptake. An alternative possibility is nickel competition at the level of iron-regulated enzymes, as reported for various Fe-S (for example, aconitase and succinate dehydrogenase) and other iron-dependent enzymes in mammalian cells [59].

Other iron-related genes whose mutation makes cells specifically sensitive to nickel or cadmium are Ccc2 (a P-ATPase responsible for copper loading of the Fe [II] oxidoreductase Fet3) and Smf3 (a divalent metal transporter that mobilizes iron ions from the vacuole to the cytosol under conditions of iron deficiency). Mutations affecting the human orthologs of these genes respectively cause Wilson disease (characterized by abnormal copper accumulation in liver) [60] and microcytic anemia with hepatic iron overload [61] (Additional data file 2).

### Metal-resistant mutants

**Figure 5:**
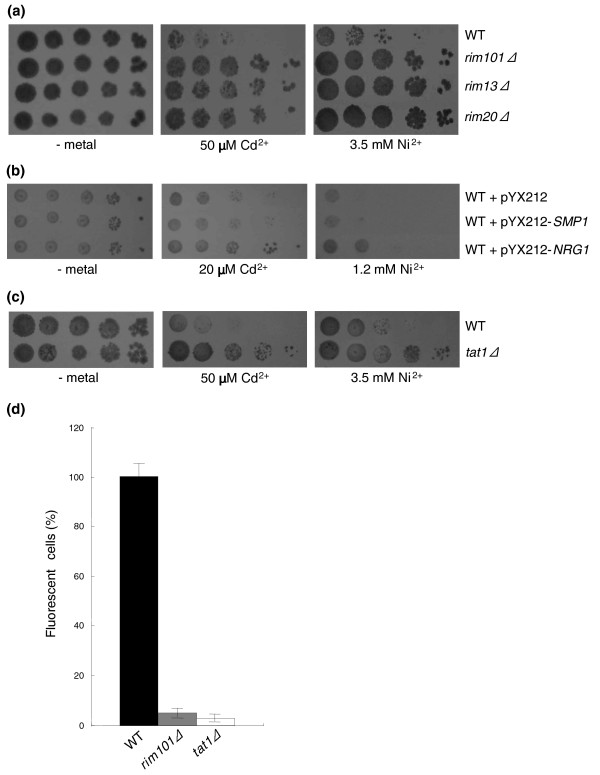
Rim101-mediated metal resistance. **(a) **Serial dilution assays documenting the cadmium and nickel resistance of *rim101Δ *and of representative Rim101-related mutants. Wild-type (WT) and mutant strains were grown in the absence of exogenously supplied metals or in the presence of the indicated concentrations of cadmium and nickel. **(b) **Over-expression of Nrg1, but not Smp1 (two transcription factors negatively regulated by Rim101), enhances tolerance to both cadmium and nickel compared with WT cells. Scaled down concentrations of cadmium and nickel were utilized for these assays, which were conducted under selective, synthetic dextrose medium conditions. **(c) **Increased cadmium/nickel tolerance of a strain disrupted in *TAT1*, a membrane transporter negatively regulated by Nrg1. **(d) **Intracellular nickel accumulation by WT, *rim101Δ*, and *tat1Δ *cells analyzed by Newport Green staining (see 'Materials and methods' for details); the percentage of fluorescent cells (average ± standard deviation of three independent experiments) is expressed relative to WT (100%).

A total of 46 mutants, not considering the 45 strains that were nickel resistant but cadmium sensitive (Figure 1a; also see the next section), exhibited increased resistance to cadmium (20 mutants, six of which were in uncharacterized ORFs), nickel (11 mutants), or both metals (15 mutants, three of which were in uncharacterized ORFs; see also Additional data file 2). The latter mutants include the transcriptional repressor Rim101 plus seven genes encoding proteins involved in the proteolytic activation and/or functionality of this regulator (Figure 5a). Originally identified as a regulator of meiotic gene expression and sporulation [62], Rim101 has also recently been implicated in the control of cell wall assembly and as a determinant of monovalent cation and alkaline pH tolerance [63-65]. Although conclusive evidence on the functional relationship between activated Rim101 and cell wall construction is still lacking, recent DNA microarray data have shed light on the transcriptional targets of Rim101. These include the transcription factors *NRG1 *and *SMP1*, which themselves act as repressors of functionally heterogeneous sets of genes [64]. To gain insight into Rim101 targets that are more closely related to cadmium/nickel resistance, we over-expressed both repressors and tested metal tolerance of the resulting transformants. As shown in Figure 5b, an increase in cadmium/nickel tolerance was observed in strains over-expressing Nrg1 but not Smp1, thus pointing to the former repressor as a downstream effector of the metal resistance phenotype brought about by Rim101 deletion. Among the targets of Nrg1 [66] is the low-affinity Trp/His transporter encoded by the *TAT1 *gene, whose deletion also enhances cadmium/nickel tolerance (Figure 5c). In addition, when tested with the fluorescent nickel chelator Newport Green [21], both *rim101Δ *and *tat1Δ *mutants exhibited strikingly reduced nickel accumulation (Figure 5d). We thus propose that Tat1 is a novel entry route for nonessential metals in yeast. Interestingly, mammalian orthologs of Tat1 encode similarly promiscuous transporters that are involved in high-affinity cationic amino acid transport but also serve as receptors for various ecotropic retroviruses such as murine leukemia virus [67].

Other transporter mutants exhibiting cadmium (but not nickel) resistance include *smf2Δ*, an intracellular manganese transporter [52] (see also Figure 7), and *zrt3Δ*, which is a transporter that mobilizes zinc ions from the vacuole to the cytoplasm [68]. Additional mutants of this kind are disrupted in the vacuolar transporter chaperones Vtc4 (nickel/cadmium resistant) and Vtc1 (nickel resistant), both of which have previously been reported to cause manganese resistance when deleted [69]. Also notable among the genes that when deleted cause cadmium and/or nickel resistance are Sif2, a subunit of the Set3C histone deacetylase complex whose disruption increases telomeric silencing, the cell cycle regulators Cln3 and Sap190, and the mitogen-activating protein kinase cascade regulator Sis2.

### Mutations in the ESCRT and in the endosome-to-Golgi retromer complexes differentially affect cadmium and nickel tolerance

**Figure 6:**
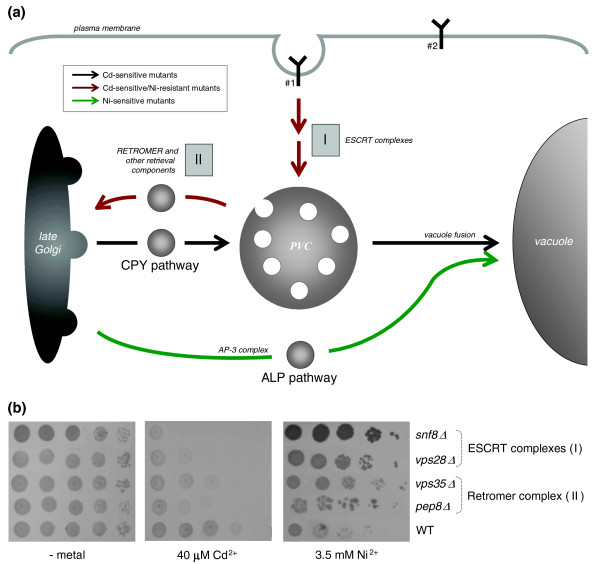
Cadmium sensitive/nickel resistant mutants and protein traffic networks centred on the vacuole and the Golgi. **(a) **Schematic representation of the endocytotic pathway, including targeting to (and formation of) the prevacuolar compartment (PVC; pathway I), and protein retrieval from the PVC to the late Golgi (pathway II). The Golgi-to-vacuole, carboxypeptidase Y (CPY) and alkaline phosphatase (ALP) pathways that, when disrupted, respectively lead to cadmium and nickel sensitivity are shown for comparison. Pathways whose disruption determines cadmium sensitivity but nickel resistance are indicated with red arrows; and pathways that cause cadmium or nickel specific sensitivity when disrupted are indicated with black and green arrows, respectively. The Y-shaped symbols indicate plasma membrane transporters whose deletion causes cadmium (#1; for example, Smf1) or nickel (#2; for example, Fur4 and Tna1) resistance; see Additional data file 2 for further details on the genes that are involved in these pathways. **(b) **Serial tenfold dilutions of mutant strains representative of pathway I and II assayed for their capacity to grow onto yeast extract-peptone-dextrose (YPD) plates supplemented with the indicated cadmium and nickel concentrations; the wild-type (WT) control strain is shown at the bottom of each panel.

**Figure 7:**
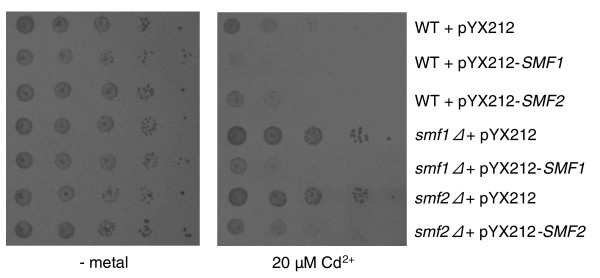
Smf transporters and cadmium toxicity. Serial dilution plate assays (synthetic dextrose medium) comparing the cadmium tolerance of *SMF1 *and *SMF2 *disrupted or overexpressing strains as indicated. Wild-type (WT) cells transformed with the empty pYX212 vector served as controls for these experiments; a no-metal control is shown in the left panel.

As was anticipated (Figure 1), mutations in 45 genes, more than half of which had never previously been implicated in metal tolerance, oppositely affect cadmium and nickel toxicities, making cells more sensitive to cadmium while increasing nickel tolerance. As shown in Figure 6a (also see Table 1 and Additional data files 3 and 4), 70% of these genes are involved in protein traffic to and formation of the prevacuolar compartment (PVC; pathway I; 20 mutants), and in protein retrieval from the PVC to the late Golgi (pathway II; ten mutants). Some of these mutants, belonging to pathway I, were previously shown to be cadmium sensitive [52,70-72] or nickel resistant [73], whereas seven pathway II mutations, only one of which known to cause cadmium sensitivity, were found to increase nickel tolerance [74]. Newly identified pathway I mutants include all class E *vps *components of the 'endosomal sorting complexes required for transport' (ESCRT I, II and III) [75,76]. Pathway II mutants are comprised of genes involved in protein retrieval to the Golgi, including all components of the 'retromer complex' and other functionally related proteins such as Vps30 and the phosphatidylinositol-3P binding nexin Snx3 [77,78]. Representative phenotypes of mutants affected in these pathways, which are conserved from yeast to humans, are shown in Figure 6b. Targeting to the PVC and formation of the 'multivesicular body' by the ESCRT pathway are involved in clearance of misfolded membrane proteins, downregulation of plasma membrane receptors and transporters, localization and processing of vacuolar components, and removal of selected regions of the plasma membrane, coupled with ingestion of surrounding small molecules, through 'fluid phase endocytosis' [75,76,79]. Pathway II, instead, is responsible for recycling hydrolase receptors and other vacuolar traffic components from the PVC to the late Golgi and to the plasma membrane [77,80,81]. Mutational inactivation of these pathways can lead, for instance, to an abnormal accumulation of plasma membrane transporters that may promiscuously internalize toxic metals (I), or to protein missorting and impaired vacuole functionality, including metal detoxification (II). Both scenarios readily apply to and explain cadmium sensitivity. This metal, in fact, is taken up and mobilized through Smf1 and Smf2 [52,82], two membrane transporters that are downregulated via the ESCRT and whose over-expression increases cadmium toxicity (Figure 7). On the other hand, cadmium is known to be detoxified by vacuolar components such as the glutathione S-conjugate transporter Ycf1, disruption of which specifically impairs cadmium tolerance [10]. Thus, a cadmium sensitivity phenotype is also expected for mutations interfering with proper sorting of these components (for example, Ycf1) or with retrieval from the PVC to the Golgi of receptors that mediate the trafficking of other components required for vacuole biogenesis and functionality.

**Figure 8:**
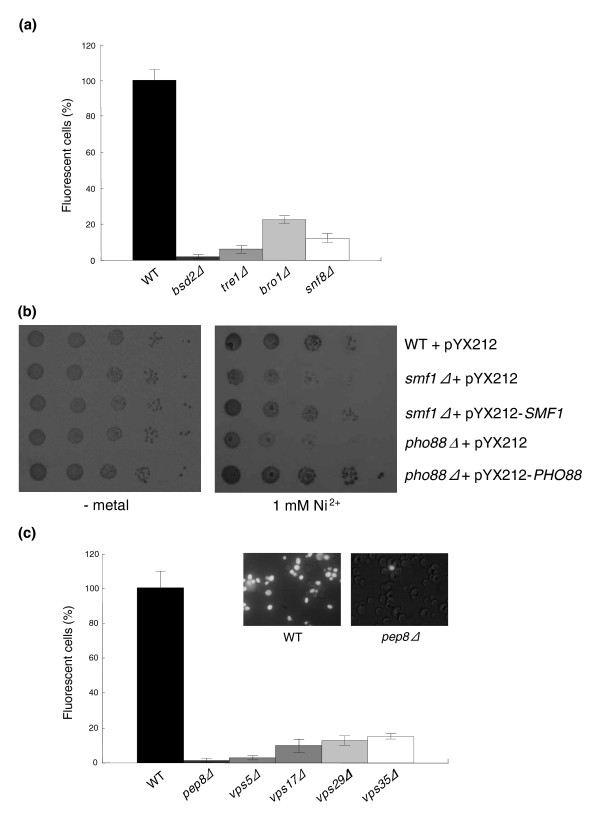
Enhanced nickel tolerance of endocytotic and retromer pathway mutant strains. **(a) **Nickel accumulation by wild-type (WT) and pathway I mutant strains (see Figure 6a). The indicated mutants were exposed to NiCl_2 _(1 mmol/l), treated with Newport Green, and visualized using fluorescence microscopy (see 'Materials and methods'). The percentage of fluorescent cells (average ± standard deviation of three independent experiments) is expressed relative to wild-type (WT; 100%). **(b) **Enhanced nickel tolerance of the Pho88 over-expressing strains. Serial dilution assays comparing the nickel tolerance of *smf1Δ *and *pho88Δ *strains transformed with the empty pYX212 vector or with the same vector bearing the *SMF1 *or the *PHO88 *coding sequences. **(c) **Nickel accumulation by WT and the indicated retromer-related (pathway II) mutant strains analyzed by Newport Green staining as in panel a; representative images of WT and mutant cells (100× magnification) are shown in the insets.

Less straightforward is the relationship between mutations in the same set of genes and resistance to nickel (outlined in Figure 6a), a metal that is also subjected to vacuolar detoxification ([83] and the present data; for example see Figure 2), but whose mechanisms of internalization (and export) are still largely unknown. As shown in Figure 8a (but also see Eide and coworkers [84]), pathway I mutants all exhibit a markedly reduced nickel accumulation, suggesting that export and/or reduced uptake may underlie the nickel resistance displayed by these mutants. Potential candidates for this role are transporters (or transport-related proteins) such as Smf1 and Pho88, which are known to interact with one or more components of pathway I [52,85] and that cause nickel sensitivity when disrupted (Additional data file 3). To test this hypothesis we assayed the nickel tolerance of the corresponding over-expressing strains, which was increased in the case of Pho88, but not Smf1 (Figure 8b). This points to an as yet unidentified role of Pho88 in nickel tolerance. It is possible, however, that other uptake systems impaired in ESCRT mutants (for example, fluid-phase endocytosis) as well as missorting to the plasma membrane of an as yet unidentified metal exporter may also contribute to nickel tolerance. Indeed, among mutations causing nickel specific resistance is Siw14, a tyrosine phosphatase that is involved in actin filament organization, whose disruption leads to a defective fluid phase endocytosis [86].

A different mode of action probably applies to the expanded set of retromer-related mutants that we also identified as nickel resistant (see pathway II in Figure 6a). One of these mutants (*vps5Δ*) was previously reported to have a nickel uptake capacity similar to that of WT in intact cells, but a threefold higher uptake rate after plasma membrane permeabilization [74]. Based on these observations, it was proposed that in this particular *vps *mutant an unidentified late Golgi Mg^2+^/H^+ ^exchanger could be missorted to the vacuole, where it would promote enhanced nickel accumulation (and detoxification). At variance with this hypothesis, we found that only a small fraction of cells mutated in various retromer-related components were able to accumulate nickel (as revealed by Newport Green fluorescence), whereas most cells were not fluorescent and thus apparently unable to accumulate nickel ions (Figure 8c). Whether this is due to a reduced uptake or to an enhanced export of nickel is not known at present. It should be noted, however, that defects in this particular traffic network can cause protein missorting to the vacuole, but also to the plasma membrane [81,87-89]. It is thus conceivable that avoidance and/or extrusion of nickel by a divalent cation transporter (or exchanger) mislocalized to the plasma membrane might be responsible for the increased nickel tolerance of these mutants. The opposite situation holds for two plasma membrane located uracil and nicotinic acid transporters, Fur4 and Tna1, which when deleted cause nickel resistance along with reduced intracellular nickel accumulation, and for which we propose a promiscuous role in nickel internalization (Additional data file 6).

Other cadmium-sensitive/nickel-resistant strains are mutated in amino acid metabolism enzymes (for example, Aat2 and Aro2) and nuclear components (for example, Mog1, Nnf2, Spt7, and Srb8), including the putative catalytic subunit of a class II histone deacetylase (Hda1), as well as in the uncharacterized ORF YIL039W. Also noteworthy are mitochondrion defective mutants, one of which (*mam3Δ*) was previously reported to be cadmium sensitive, but resistant to cobalt and zinc [90].

### Cellular toxicity signatures of other metals

**Figure 9:**
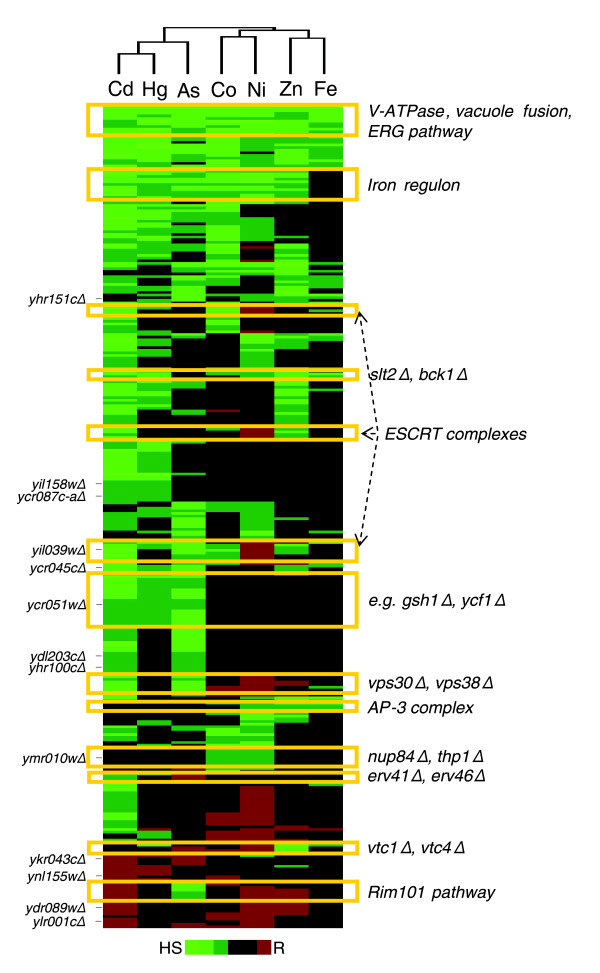
Multimetal toxicity signatures. Hierarchical clustering of cadmium and nickel tolerance-modulating mutations with the phenotypic profiles of other metals. Cadmium/nickel sensitive or resistant strains were exposed in triplicate to HgCl_2 _(190 μmol/l), NaAsO_2 _(1.5 mmol/l), CoCl_2 _(2 mmol/l), ZnCl_2 _(18 mmol/l), and FeCl_3 _(15 mmol/l), followed by serial dilution assay verification of mutations affecting cell tolerance to this expanded set of metals (see 'Materials and methods' for details). The x-axis corresponds to the metals examined, and the y-axis indicates gene deletions. Mutants exhibiting either an enhanced sensitivity or resistance, or no phenotype are represented in green, red and black, respectively. Metal tolerance (from 'high sensitivity' [HS] to 'resistance' [R]) of the different mutant strains is indicated in a false color scale; only strains sensitive or resistant to at least two metals are shown (see Additional data file 7 for the entire database of multimetal phenotypes). Hierarchical clustering analysis was performed with EPCLUST, as specified in the legend to Figure 3, leaving out 67 cadmium-specific and nine nickel-specific gene mutations (listed in Additional data file 7). Representative genes and pathways affecting multimetal tolerance as well as a subset of co-clustering uncharacterized open reading frames with orthologous sequences in other organisms (see Additional data file 2) are indicated on the right-hand and left-hand, respectively.

As a last step in our analysis, we considered the extent to which the range of genes and pathways that, when disrupted, affect cadmium/nickel tolerance overlaps that of other metals. To this end, the entire set of sensitive and resistant mutant strains was exposed to sublethal concentrations of four additional metals with varying degrees of chemical (and/or biologic effect) similarity to cadmium and nickel, plus the metalloid AsO_2_^-^. As shown by the clustering analysis in Figure 9, which does not include 67 cadmium-specific and nine nickel-specific mutants (Additional data file 7), the overlap was higher for sensitivity-conferring than for resistance-conferring mutations, and various pathways involved in multimetal sensitivity could be identified.

The closest resemblance with cadmium was observed for mercury, a highly toxic, thiophilic group IIb metal with a nearly identical atomic radius. This is followed by arsenite, which despite its chemical dissimilarity is known to bind thiols (especially dithiols [91]) and to share various cellular toxicity similarities with cadmium [32]. The most prominent divergence between cadmium and arsenite pertains to the Rim101 pathway (which when disrupted causes AsO_2_^- ^sensitivity but resistance to cadmium, nickel and zinc) and to a few mutants (which exhibited the opposite phenotypic response, such as *erv41Δ *and *erv46Δ*). Interestingly, the same metal discrimination capacity applies to the Rim101/Nrg1-regulated plasma membrane transporter Tat1, whose disruption leads to resistance to cadmium, nickel and zinc, but not arsenite.

The phenotypic overlap between nickel and cobalt was not as high as one might have expected based on their chemical similarity. Especially noteworthy is the increased sensitivity to cobalt and to all the other tested metals, except nickel, exhibited by ESCRT pathway mutants, and the only partial overlap between nickel and cobalt observed for retromer mutants. Also worthy of note are the different metal tolerance phenotypes associated with the Fur4 and Tna1 transporters, whose deletion causes sensitivity to cadmium and to other metals, but nickel resistance (see Additional data file 6 for representative phenotypes). One of them (Tna1) causes resistance to both nickel and cobalt when disrupted, whereas deletion of the other transporter (Fur4) makes cells resistant to nickel and zinc but not cobalt. Conversely, disruption of the Smf1 transporter as well as disruption of various components of the Adaptor Protein complex AP-3 involved in the ALP pathway (Table 1 and Additional data file 3) causes nickel but not cobalt sensitivity.

As predicted by the protective effect exerted on both WT and mutant cells (Figure 4) and by its 'hard' Lewis acid nature, Fe(III) was the most divergent of the metals investigated. Also apparent in Figure 9 is that under conditions of iron sufficiency, mutations in genes belonging to the iron regulon cause increased sensitivity to all of the examined metals except iron itself. This suggests that, albeit with different mechanisms, toxic metal-induced iron depletion may be a common feature of many (if not all) toxic metals. Zinc and Fe(III), both of which are essential metal ions, clustered together despite their chemical dissimilarity. On the whole, however, we find a fairly close overlap between the chemical properties and the cellular toxicity signatures of the various metals. For example, clustering based on the phenotypic profiles of a selected subset of mutants fits with chemical properties such as the ability of the various metals to form insoluble sulfides in a strongly acidic environment, which is shared by cadmium, mercury and As(III), but not nickel, cobalt, zinc, or Fe(III). As further shown in Figure 9, multimetal phenotypic profiling also allowed to annotate various ORFs, some of which are homologous to disease-related human genes. For instance, YCR045C and YCR051W are homologous, respectively, to Pcsk9 and Tnks2. Pcsk9 is an endoplasmic reticulum serine protease that is involved in an autosomal dominant form of hypercholesterolemia [92], a disease that is also induced by dietary metal imbalance [93]; Tnks2 is a cytosolic member of the poly(ADP)ribose polymerase family, whose over-expression affords cytoprotection by preventing excessive poly(ADP)ribose polymerase activation and NAD depletion after exposure to DNA-damaging agents [94].

## Conclusion

As revealed by this study, which interrogated all nonessential genes of *S. cerevisiae *for their role in modulating metal toxicity, 16 functional subnetworks (comprised of 207 genes, at least half of which had never been implicated in metal tolerance previously) negatively influence cadmium and/or nickel tolerance when disrupted. Core genes influencing cadmium and nickel tolerance were mapped to nine subnetworks, a subset of which (for example, V-ATPase, vacuole fusion, and the ERG pathway) cause enhanced co-sensitivity to mercury, arsenite, cobalt, zinc and iron, thus pointing to the occurrence of multimetal defense systems in yeast. Seven of these subnetworks were expanded to include additional mutations specifically associated with cadmium and/or nickel sensitivity, along with six additional subnetworks causing metal-specific (especially cadmium-specific) sensitivity (Table 1). Only one of the latter subnetworks (with a bearing on the ALP branch of the Golgi-to-vacuole traffic pathway) was found to be specifically involved in nickel tolerance, as opposed to five subnetworks causing cadmium-specific sensitivity when disrupted. Thus, cadmium is not only more toxic than nickel, but it also has a broader spectrum of cellular processes that directly or indirectly contribute to its detoxification. Most prominent among these processes are those related to vesicular protein traffic (including the endocytotic pathway and a different branch of the Golgi-to-vacuole traffic), antioxidant defense, and DNA repair. The latter, in particular, further strengthens the causal relationship between cadmium genotoxicity and DNA repair [6]. In fact, although cadmium and nickel have both been recognized as human carcinogens [2,3], mutagenic activity appears to be a distinguishing feature of cadmium [1,4]. Nickel, instead, is a weak mutagen with a marked nuclear tropism, whose carcinogenicity is thought to primarily rely on unprogrammed chromatin modification [5]. It is interesting to note in this regard that the nuclear pore complex is one of the few core subnetworks enriched in nickel-specific sensitive mutants. Also interesting is that three out of eight mutants specifically resistant to nickel (but unrelated to vesicular traffic; Additional data file 7) are deleted in genes encoding distinct chromatin modification enzymes (*HDA1*, *EAF7*, and *SPT7*) and one is deleted in a Ran homolog of the Ras GTPase family (*MOG1*) that is involved in protein traffic through the nuclear pore.

Many metal toxicity-modulating pathways are related to metal damage prevention or repair, whereas others appear to play a more general (and indirect) role in promoting cell survival/recovery under stress conditions. Especially noteworthy among the latter are mRNA decay and nucleocytoplasmic transport, two processes that to our knowledge have not previously been implicated in metal tolerance and that might explain the variety of putative target genes previously identified as cadmium stress responsive [11]. Their identification among metal sensitivity-conferring mutations suggests that not only the clearance of damaged (or unwanted) proteins by the proteasome and transcriptional regulation, but also mRNA turnover and relocalization are important for translational/metabolic reprogramming under conditions of metal stress. Interestingly, coordinate downregulation of iron-related proteins mediated by mRNA degradation under iron starvation conditions [95] as well as mRNA mistranslation after chromium exposure [96] have recently been described in yeast. The fact that structurally diverse yet functionally related gene products cause metal sensitivity when disrupted provides strong evidence that the cellular processes represented in specific subnetworks play an important role in preventing or repairing metal-induced cell damage. However, this does not exclude the possibility that a subset of the mutant strains that we have identified as metal sensitive are due to chemical-genetic synthetic lethality resulting from direct attack (and inactivation) of a functionally related protein target by the metal. Mutations associated with this kind of metal-induced lethality are likely to be enriched in the genes we classified as 'solitary'.

Among the unrelated stressors we examined, alkaline pH emerged as the most closely related to cadmium/nickel stress. This genomic phenotyping resemblance was traced back to iron deficiency, which - albeit with different mechanisms - is caused by both cadmium and nickel and appears to be a fairly general effect of metal toxicity (Figure 9). Broad-range transporters were identified as the most proximal effectors of iron deficiency-related and other kinds of altered metal tolerance. The latter include Tat1, which is a low-affinity Trp/His transporter negatively regulated by Nrg1 [66], which emerged from this study as one of the downstream effectors of the multimetal resistance caused by disruption of the Rim101 pathway. Toxic metal internalization (or abnormal intracellular mobilization) thus appears to be one of the most general and detrimental effects caused by transporter promiscuity, a trait that has probably evolved as a way to deal with multiple nutritional deficiencies under nutrient limiting (but toxic metal-free) conditions. This provides novel mechanistic support to the notion that nutrient limitation (especially iron and copper, but also amino acids and vitamins) may aggravate metal toxicity in malnourished human populations. Another outcome of this study was the identification of 24 uncharacterized ORFs that are involved in metal tolerance, which lend themselves as novel candidate genes that are worthy of further investigation.

Systematic comparison of the cellular toxicity signatures of cadmium and nickel with those of five additional metals revealed significant overlap between their chemical and cellular toxicity properties. However, it also uncovered an unexpected degree of metal specificity, especially regarding mutations that cause resistance to nickel but sensitivity to most other metals. The hot spots for such mutations were mapped to the ESCRT and the retromer complexes, thus pointing to the ability of these pathways to discriminate between otherwise similar metals and to the potential use of selected toxic metals (for example, cadmium and nickel) as chemical probes of intracellular traffic functionality.

## Materials and methods

### Yeast strains and culture conditions

The strains used in this study derive from the *S. cerevisiae *Genome Deletion Project [18]. They were purchased from Open Biosystems (Huntsville, AL, USA) and converted into a 384-well plate format by manual multipinning. Deletion of individual nonessential genes (or ORFs) were in the MATα BY4742 background, except for 79 strains with a MATα BY4739 parental background. Untagged deletion strains utilized for phenotype verification were in the parent MATα BY4741 background and were obtained from EUROSCARF (Frankfurt, Germany). Cells were grown at 30°C on yeast extract-peptone-dextrose (YPD) or synthetic dextrose medium (supplemented with leucine, lysine, and histidine, but without uracil), as indicated. For some experiments, YPD was supplemented with FeCl_3 _or adjusted to pH values ranging from 6 to 8.5 with the addition of 50 mmol/l MOPS (3- [4-morpholino]propane sulfonic acid) or TAPS (N- [tris(hydroxymethyl)methyl]-3-aminopropanesulfonic acid), as specified in the text.

### Metal tolerance screening

A total of 4,688 single gene deletion mutants, not including 90 strains that failed quality control and 48 slow-growth strains previously shown to exhibit a high false-positive rate [97], were utilized for genomic phenotyping. Metal titrations coupled with serial dilution spot assays (starting from cultures pregrown in YPD at an optical density at 600 nm [OD_600_] of 1.0 and diluted up to 10,000 fold in tenfold increments before spotting; see below) were initially carried out in the parent WT strains to determine metal concentrations, allowing for about 90% of control ('minus metal') growth after 48 hours. These concentrations ranged from 40 to 60 μmol/l and from 2.5 to 4.5 mmol/l for cadmium and nickel, respectively. Metal concentrations for genomic phenotyping were further refined in pilot experiments carried out with an arrayed test set of 384 strains, including the WT strains plus two known cadmium-sensitive mutants (*yap1Δ *[7] and *ycf1Δ *[10]) and one nickel-sensitive mutant (*pep5Δ *[98]) as positive controls. Optimal concentrations of 50 μmol/l cadmium and 2.5 mmol/l nickel were thus determined and utilized for the screening.

To this end, individual plates from the deletion strain collection (384-well format master plates with eight empty wells as contamination controls) were inoculated into 150 μl liquid YPD plus 200 μg/ml G418 (GIBCO-Invitrogen, Carlsbad, CA, USA) using a 384-pin tool (VP 384F; V&P Scientific Inc., San Diego, CA, USA). After 2 days at 30°C, cells were replica inoculated onto YPD-agar without G418, supplemented or not supplemented with the appropriate metal. This was done with the use of a library copier (VP 381; V&P Scientific Inc.) by touching the bottom of the wells and then raising and lowering the multipin replicator three times, in order to mix the cells and obtain a properly diluted inoculum (about 500 cells/pin). After 2 to 3 days at 30°C, plates were examined for metal-sensitive and metal-resistant strains according to relative colony size, followed by digital image recording. A positive result was scored when colony size under metal-supplemented conditions was diminished (no growth or slow growth phenotype in the case of metal sensitivity) or augmented (overgrowth phenotype in the case of metal resistance) compared with neighboring (unaffected) strains and with the colony size of the corresponding strains grown on the 'minus metal' plate (Additional data file 1 [Figure S1A]). Five replicate screens starting from fresh liquid cultures were run for each metal (Additional data file 2 provides details on the cumulative outcome of this multireplicate screening), followed by verification of strains that were scored as sensitive or resistant in at least three screens by serial dilution spot assays (see below).

### Validation and multimetal assays

Strains that were deemed as positive (sensitive or resistant) in the primary screen as well as strains consistently exhibiting an overgrowth or slow-growth phenotype in control ('minus metal') plates were individually verified by serial dilution spot assays. Mutant strains of interest were recovered from the original 96-well plates, assembled into a new plate, and cultured in YPD medium as above. After 24 hours at 30°C, the OD_600 _of individual cultures was determined with a microplate reader, adjusted with YPD medium to an OD_600 _value of 1.0 and serially diluted in tenfold increments. Aliquots (4 μl) of each dilution were spotted onto YPD-agar plates in the presence or absence of appropriate metal concentrations (40 μmol/l CdCl_2 _and 2.5 mmol/l NiCl_2 _for sensitive strains, and 50 μmol/l CdCl_2 _and 3.5 mmol/l NiCl_2 _for strains scored as resistant) and growth was examined after incubation at 30°C for 2 to 3 days. Mutant strains exhibiting a reduction in growth at the first, second, or third (or fourth) dilution were classified as having a 'high', 'medium', or 'low' metal sensitivity (HS, MS, and LS, respectively); only one type of metal-resistant phenotype was recorded (Additional data file 1 [Figures S1B and S1C]). The rate of validation of the phenotypes determined in primary screens was 85% for cadmium and 81% for nickel. Appropriately lower metal concentrations (15 to 20 μmol/l cadmium and 1 to 1.2 mmol/l nickel for sensitivity and resistance, respectively) were used for assays carried out in synthetic dextrose medium (see below). Eighteen strains identified as metal-sensitive in our screen correspond to 'dubious' ORFs [99] overlapping '*bona fide*' ORFs that were also found to be metal sensitive. The physical and phenotypic overlapping of this subset of ORFs is annotated in Additional data file 2, from which all redundant ORFs were removed. An additional four strains deleted in 'dubious' ORFs overlapping the 5'-end of ORFs not present in the mutant collection were replaced with the latter ORFs and included in the final dataset. Identical screening and validation assay conditions were applied to the 388 cadmium/nickel sensitive or resistant strains that were tested with four additional metal, plus the metalloid AsO_2_^-^. The following concentrations, determined by metal titrations coupled with serial dilution spot assays carried out on WT cells (as described above for cadmium and nickel), were utilized: 190 μmol/l HgCl_2_, 1.5 mmol/l NaAsO_2_, 2 mmol/l CoCl_2_, 18 mmol/l ZnCl_2_, and 15 mmol/l FeCl_3_.

### Overexpression studies

The *Escherichia coli *XL1-Blue strain was used for DNA cloning experiments. The coding sequences of the genes of interest (*NRG1*, *PHO88, SMF1*, *SMF2*, and *SMP1*; see the text for further details) were obtained by polymerase chain reaction, using genomic DNA from the BY4742 strain as template and the forward and reverse oligonucleotide primers summarized in Table 2.

**Table 2:** Oligonucleotide primers used for DNA amplification

Gene name	Forward/reverse	Primer
*NRG1*	Forward	5'-(CTCGGTCCGCCACCATGTTTTACCCATATAACTATAGTAAC)-3'
*NRG1*	Reverse	5'-(CTCGGACCGTTATTGTCCCTTTTTCAAATGTGTTC)-3'
*PHO88*	Forward	5'-(CGCGGTCCGCTACGTAGCCACCATGAATCCTCAAGTCAGTAACATC)-3'
*PHO88*	Reverse	5'-(CGCGGACCGTCATTCAGCCTTAACACCAGCG)-3'
*SMF1*	Forward	5'-(CGCGGTCCGGTTTAAACAGGCCACCATGGTGAACGTTGGTCCTTCTC)-3'
*SMF1*	Reverse	5'-(CGCGGACCGTTAACTGATATCACCATGAGACATG)-3'
*SMF2*	Forward	5'-(CGCGGTCCGCTACGTAGCCACCATGACGTCCCAAGAATATGAACC)-3'
*SMF2*	Reverse	5'-(CGCGGACCGTTAGAGGTGTACTTCTTTGCCCG)-3'
*SMP1*	Forward	5'-(CTCGGTCCGCCACCATGGGTAGAAGAAAAATTGAAATTGAACC)-3'
*SMP1*	Reverse	5'-(CTCGGACCGTTAATCTGGAGAGTTTGTCGAACTCG)-3'

Individual amplicons were cloned into a modified (*Cpo*I restriction site-containing) pYX212 vector. Following sequence verification, individual constructs were utilized for yeast transformation using the lithium acetate procedure [100].

### Newport Green staining

Yeast cells were grown at 30°C to saturation, diluted to an OD_600 _of 0.3 and exposed to 1 mmol/l NiCl_2 _for 18 hours. Cells were then washed three times with phosphate-buffered saline (PBS) before being incubated for 30 minutes at 37°C in 1 ml PBS containing 7 μmol/l Newport Green DCF and 0.2% F-127 pluronic acid (Invitrogen-Molecular Probes, Eugene, OR, USA), followed by a further 30 minutes of incubation at room temperature. After an additional wash with PBS, cells were visualized by fluorescence microscopy using a Zeiss fluorescent microscope (argon laser; 488 nm). After visualization and cell counting, the fraction of fluorescent cells was determined in selected mutant strains (specified in the text) and in control WT cells.

### Data analysis

Putative human homologs were identified with BLASTP searches and through the Princeton Protein Orthology Database [101]. Information on human disease-related homologs was retrieved from the Online Mendelian Inheritance in Man database [102], the *Saccharomcyes *Genome Database [99], and by manual curation. Biologic processes associated with metal toxicity-modulating genes were identified and evaluated for statistical significance (*P*-value) with the GO Term Finder program [99]. Enrichment ratios were calculated by comparing the representation of each GO term within individual sets of metal tolerance-modulating genes with their representation in the yeast genome. Interactions between metal tolerance-modulating genes were identified computationally using the Network Visualization System Osprey [103] and visualized as specified in the BioGRID database (version 2.035 release) [22] and in the Osprey reference manual. Subnetworks were defined as a minimum of three interacting gene products sharing at least one GO biologic process annotation and connected by at least two physical (two-hybrid, affinity capture-western, affinity capture-MS, or reconstituted complex) or genetic (synthetic lethality, synthetic growth defect, synthetic rescue, dosage rescue, phenotypic suppression, or phenotypic enhancement) interactions. *P*-values for individual subnetworks were determined by a one-tailed test based on the hypergeometric distribution, using the lowest possible 'child term' (the one yielding the lowest *P*-value) allowed by the present GO categorization. Random samplings of N proteins (where N is the number of toxicity-modulating gene products in the three sets of metal sensitivity-conferring mutations [cadmium/nickel, nickel, and cadmium]) were performed 10,000 times using a script written in Perl and the above-mentioned criteria, leaving out essential genes. They respectively yielded averages of 1.2, 2.1 and 9.5 subnetworks, as compared with the 9, 11 and 16 subnetworks identified with the cadmium/nickel (79 proteins), nickel (118 proteins), and cadmium (303 proteins) sets.

## Abbreviations

ALP, alkaline phosphatase; ESCRT, endosomal sorting complexes required for transport; GO, Gene Ontology; GSH, reduced glutathione; HS, high sensitivity; LS, low sensitivity; MS, medium sensitivity; OD_600_, optical density at 600 nm; ORF, open reading frame; PBS, phosphate-buffered saline; PVC, prevacuolar compartment; WT, wild-type; YPD, yeast extract-peptone-dextrose.

## Authors' contributions

RR performed genome phenotyping screens, serial dilution, over-expression and Newport Green staining assays as well as data analysis. GM performed genome phenotyping screens and serial dilution assays. SO conceived the study and wrote the paper.

## Additional data files

The following additional data are available with the online version of this paper. Additional data file 1 provides representative primary screen data and serial dilution growth assays. Additional data file 2 shows detailed phenotypes and related information on the genes whose disruption affects metal tolerance. Additional data file 3 shows interaction subnetworks for gene products whose disruption causes nickel specific sensitivity. Additional data file 4 shows interaction subnetworks involved in cadmium-specific sensitivity Additional data file 5 illustrates broad-range metal uptake system mutants that affect cadmium tolerance. Additional data file 6 shows enhanced nickel tolerance conferred by disruption of the Tna1 and Fur4 transporters. Additional data file 7 provides the multimetal screen database.

## Supplementary Material

Additional data file 1 Presented is a composite figure showing representative examples of genomic phenotyping screens and related serial dilution assays utilized to assess metal resistance as well as the severity of metal sensitivity phenotypes. Click here for file

Additional data file 2 This file reports the name, phenotype and 'scoring rate', description, cadmium stress microarray and nickel ionome data when available, best hit, human ortholog and related disease (if applicable), as well as the GO ID, of metal tolerance-modulating genes. Click here for file

Additional data file 3 This figure shows the interaction subnetworks among gene products whose disruption causes nickel-specific sensitivity. Click here for file

Additional data file 4 This figure shows the interaction subnetworks among gene products whose disruption causes cadmium-specific sensitivity. Click here for file

Additional data file 5 This figure documents the altered cadmium tolerance of the *fet4Δ*, *smf1Δ*, and *rox1Δ *mutant strains. Click here for file

Additional data file 6 This figure documents the altered nickel tolerance of the *fur4Δ *and *tna1Δ *mutant strains. Click here for file

Additional data file 7 this document reports the metal sensitivity or resistance phenotypes of the 388 mutant strains with an altered cadmium/nickel tolerance, exposed to sublethal concentrations of mercury, arsenite, cobalt, zinc, and iron. Click here for file
