# Supplementary material for: Membrane transporters and protein traffic networks differentially affecting metal tolerance: a genomic phenotyping study in yeast
Source: Genome Biol. 2008 Apr 7;9(4):R67. doi: 10.1186/gb-2008-9-4-r67 (PMC2643938; doi:10.1186/gb-2008-9-4-r67)
Supplement: Additional data file 1 — Presented is a composite figure showing representative examples of genomic phenotyping screens and related serial dilution assays utilized to assess metal resistance as well as the severity of metal sensitivity phenotypes. [file gb-2008-9-4-r67-S1.ppt]

## Slide 1
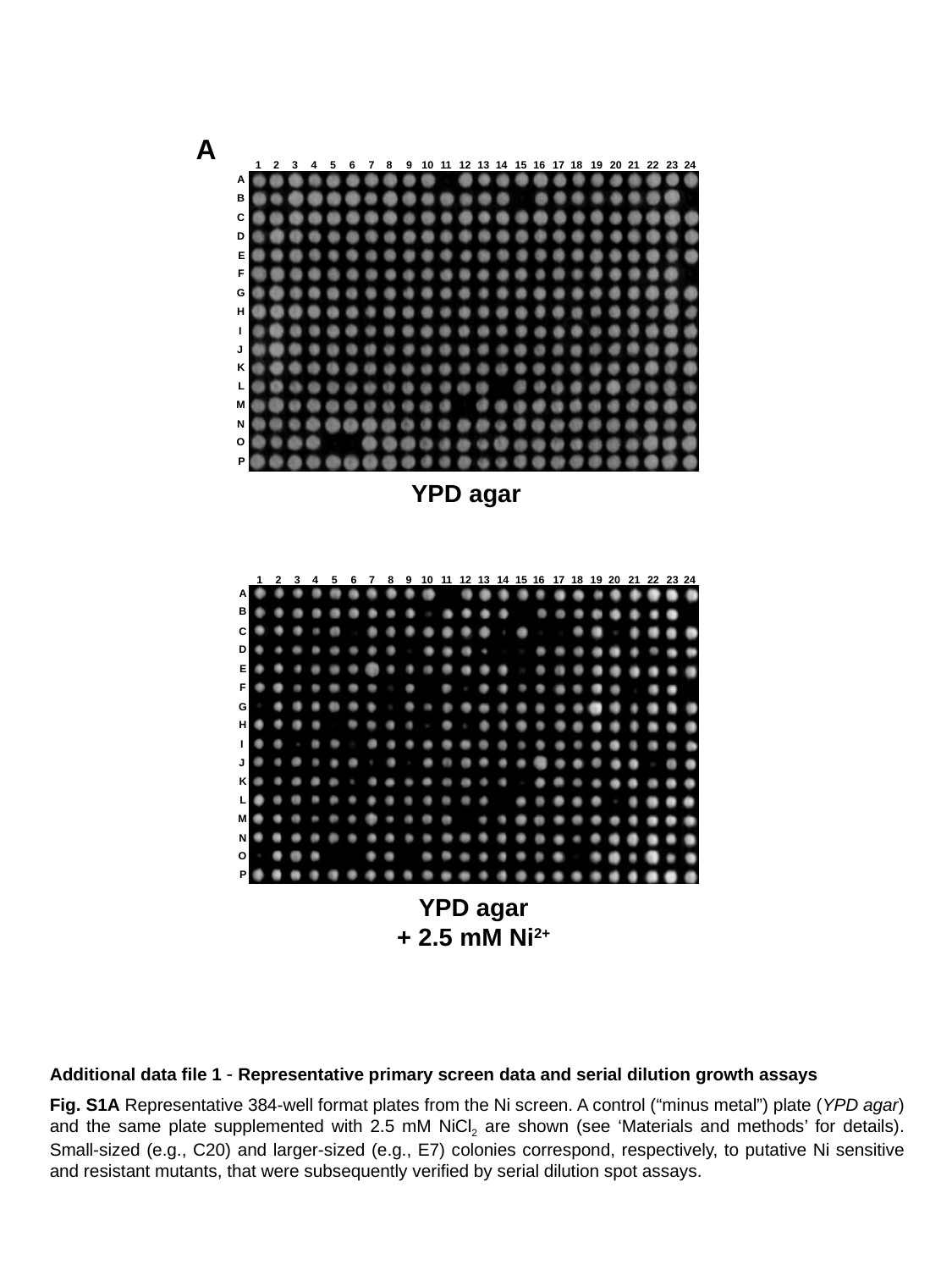

A
1
2
3
4
5
6
7
8
9
10
11
12
13
14
15
16
17
18
19
20
21
22
23
24
A
B
C
D
E
F
G
H
I
J
K
L
M
N
O
P
YPD agar
1
2
3
4
5
6
7
8
9
10
11
12
13
14
15
16
17
18
19
20
21
22
23
24
A
B
C
D
E
F
G
H
I
J
K
L
M
N
O
P
YPD agar
+ 2.5 mM Ni2+
Additional data file 1 - Representative primary screen data and serial dilution growth assays
Fig. S1A Representative 384-well format plates from the Ni screen. A control (“minus metal”) plate (YPD agar) and the same plate supplemented with 2.5 mM NiCl2 are shown (see ‘Materials and methods’ for details). Small-sized (e.g., C20) and larger-sized (e.g., E7) colonies correspond, respectively, to putative Ni sensitive and resistant mutants, that were subsequently verified by serial dilution spot assays.

## Slide 2
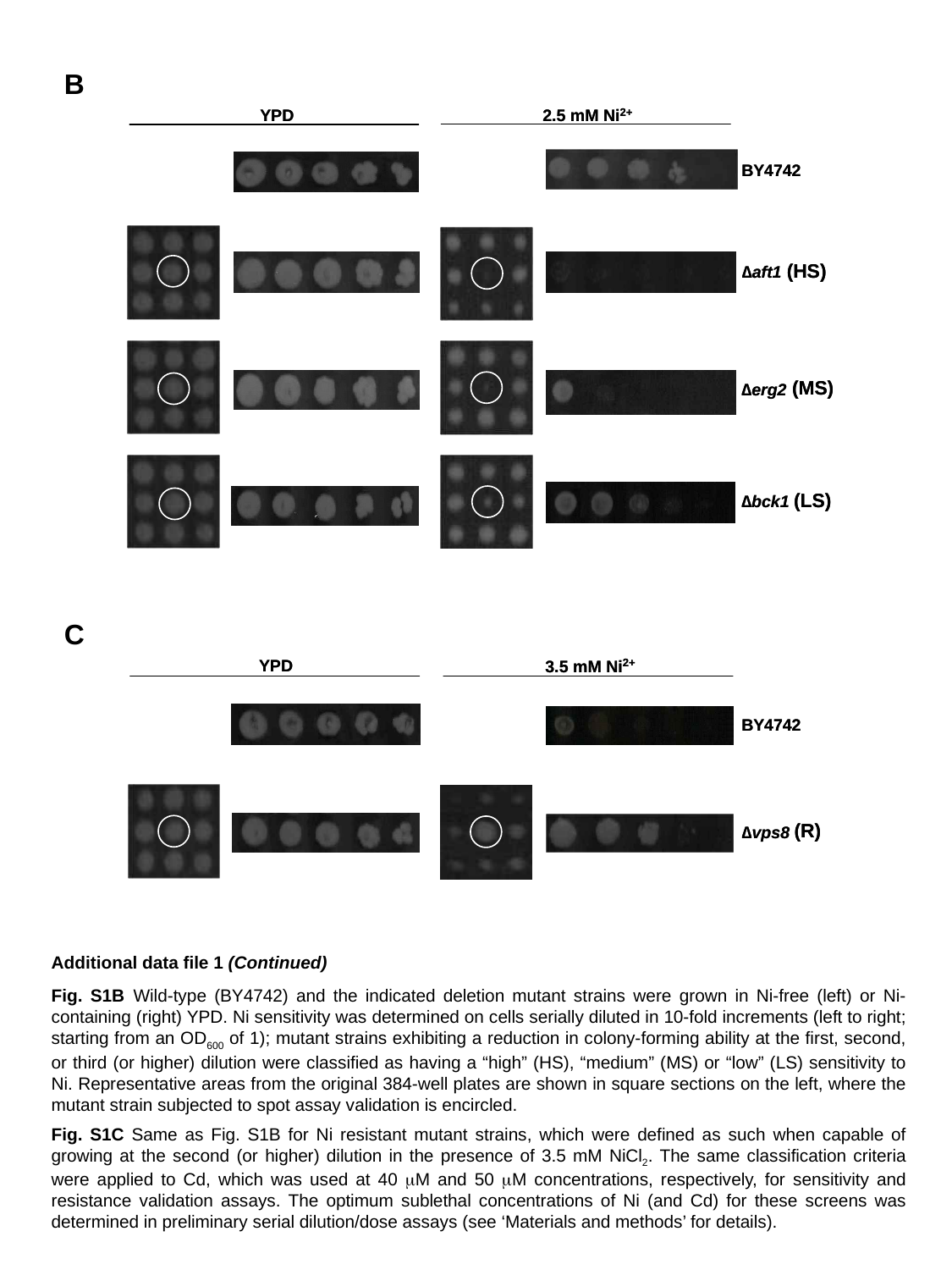

B
C
Additional data file 1 (Continued)
Fig. S1B Wild-type (BY4742) and the indicated deletion mutant strains were grown in Ni-free (left) or Ni-containing (right) YPD. Ni sensitivity was determined on cells serially diluted in 10-fold increments (left to right; starting from an OD600 of 1); mutant strains exhibiting a reduction in colony-forming ability at the first, second, or third (or higher) dilution were classified as having a “high” (HS), “medium” (MS) or “low” (LS) sensitivity to Ni. Representative areas from the original 384-well plates are shown in square sections on the left, where the mutant strain subjected to spot assay validation is encircled.
Fig. S1C Same as Fig. S1B for Ni resistant mutant strains, which were defined as such when capable of growing at the second (or higher) dilution in the presence of 3.5 mM NiCl2. The same classification criteria were applied to Cd, which was used at 40 μM and 50 μM concentrations, respectively, for sensitivity and resistance validation assays. The optimum sublethal concentrations of Ni (and Cd) for these screens was determined in preliminary serial dilution/dose assays (see ‘Materials and methods’ for details).
