# Supplementary material for: Membrane transporters and protein traffic networks differentially affecting metal tolerance: a genomic phenotyping study in yeast
Source: Genome Biol. 2008 Apr 7;9(4):R67. doi: 10.1186/gb-2008-9-4-r67 (PMC2643938; doi:10.1186/gb-2008-9-4-r67)
Supplement: Additional data file 3 — This figure shows the interaction subnetworks among gene products whose disruption causes nickel-specific sensitivity. [file gb-2008-9-4-r67-S3.ppt]

## Slide 1
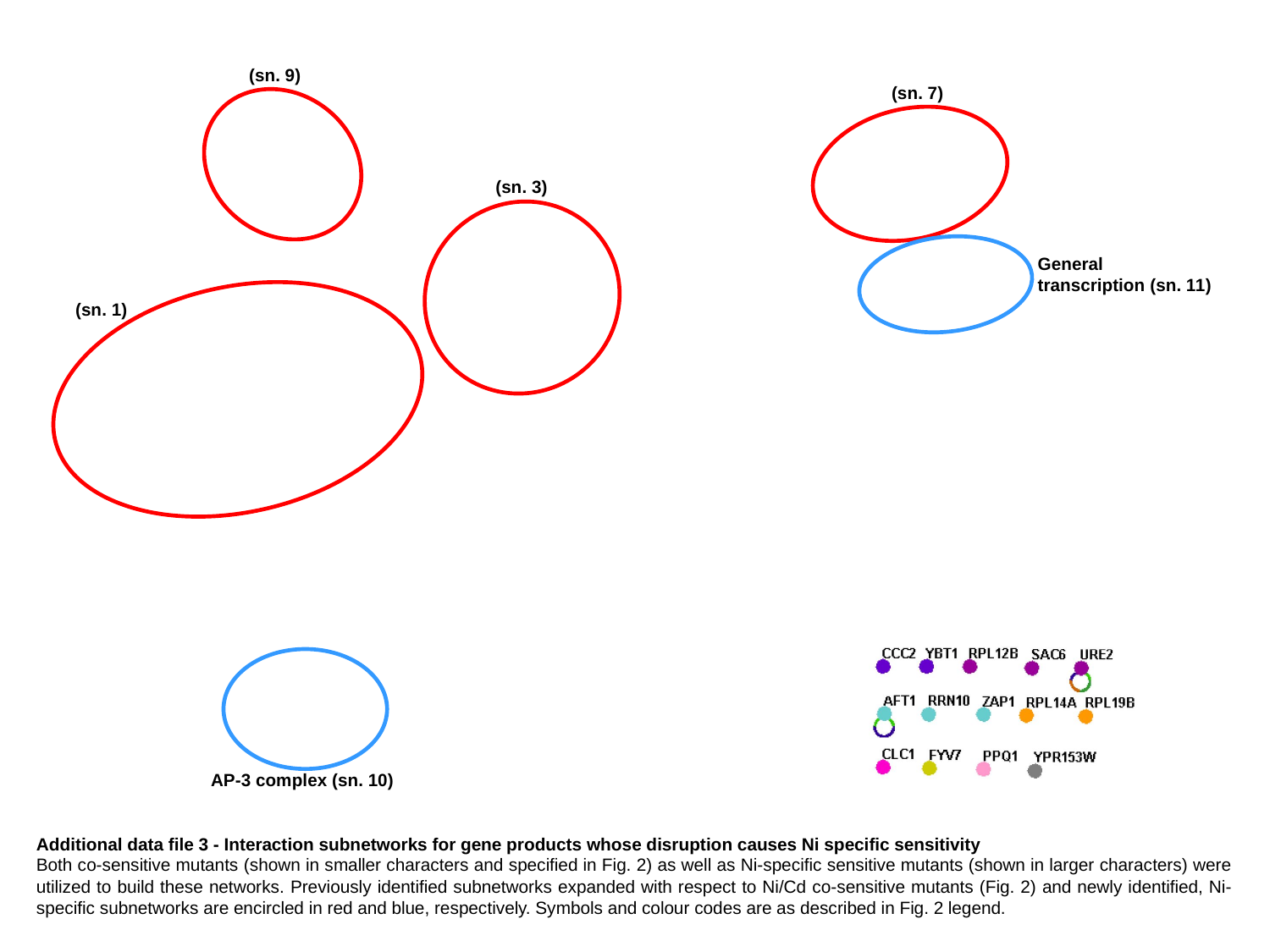

Additional data file 3 - Interaction subnetworks for gene products whose disruption causes Ni specific sensitivity
Both co-sensitive mutants (shown in smaller characters and specified in Fig. 2) as well as Ni-specific sensitive mutants (shown in larger characters) were utilized to build these networks. Previously identified subnetworks expanded with respect to Ni/Cd co-sensitive mutants (Fig. 2) and newly identified, Ni-specific subnetworks are encircled in red and blue, respectively. Symbols and colour codes are as described in Fig. 2 legend.
