# Supplementary material for: Membrane transporters and protein traffic networks differentially affecting metal tolerance: a genomic phenotyping study in yeast
Source: Genome Biol. 2008 Apr 7;9(4):R67. doi: 10.1186/gb-2008-9-4-r67 (PMC2643938; doi:10.1186/gb-2008-9-4-r67)
Supplement: Additional data file 4 — This figure shows the interaction subnetworks among gene products whose disruption causes cadmium-specific sensitivity. [file gb-2008-9-4-r67-S4.ppt]

## Slide 1
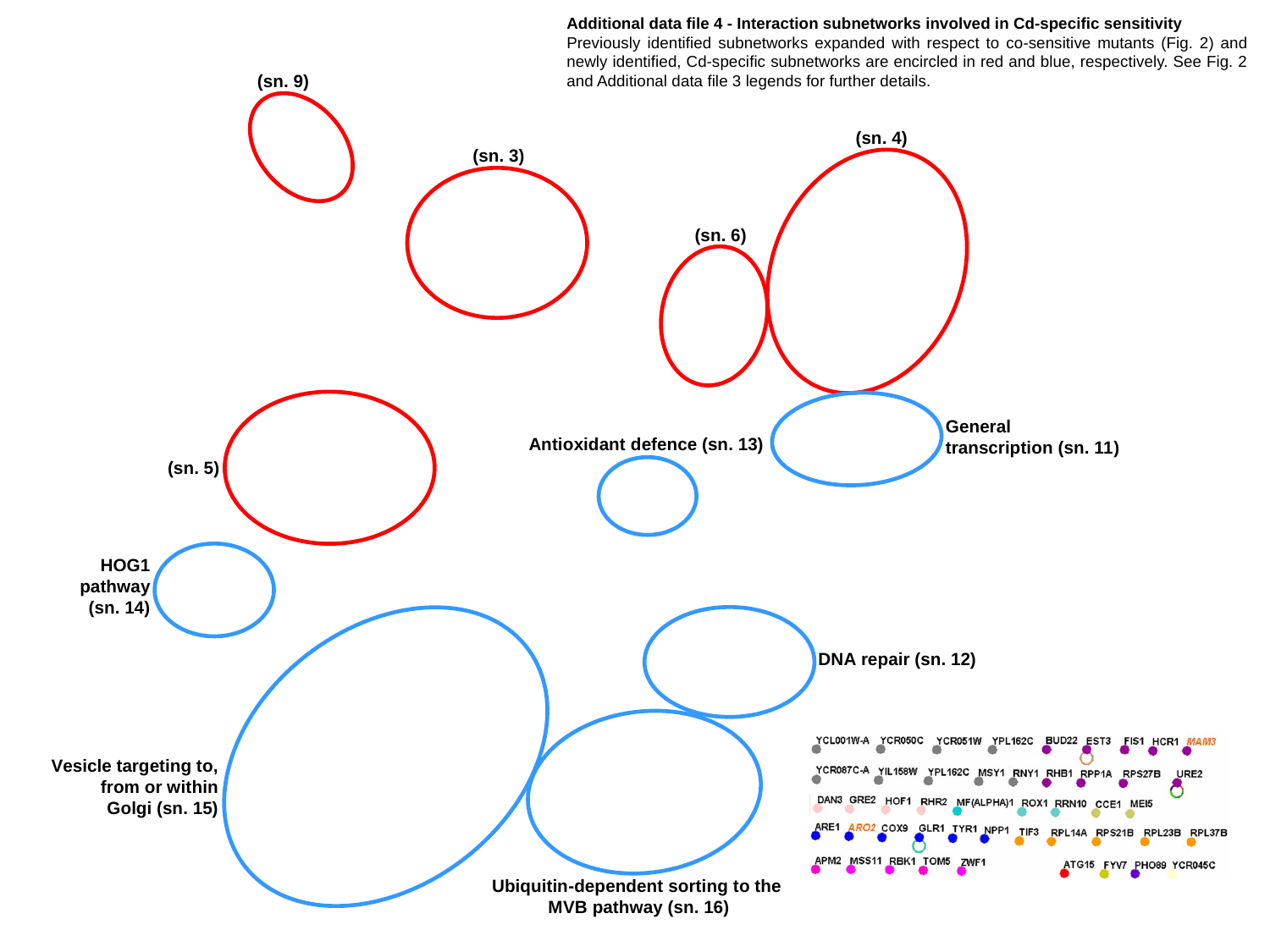

Additional data file 4 - Interaction subnetworks involved in Cd-specific sensitivity
Previously identified subnetworks expanded with respect to co-sensitive mutants (Fig. 2) and newly identified, Cd-specific subnetworks are encircled in red and blue, respectively. See Fig. 2 and Additional data file 3 legends for further details.
