# Supplementary material for: Membrane transporters and protein traffic networks differentially affecting metal tolerance: a genomic phenotyping study in yeast
Source: Genome Biol. 2008 Apr 7;9(4):R67. doi: 10.1186/gb-2008-9-4-r67 (PMC2643938; doi:10.1186/gb-2008-9-4-r67)
Supplement: Additional data file 5 — This figure documents the altered cadmium tolerance of the fet4Δ, smf1Δ, and rox1Δ mutant strains. [file gb-2008-9-4-r67-S5.ppt]

## Slide 1
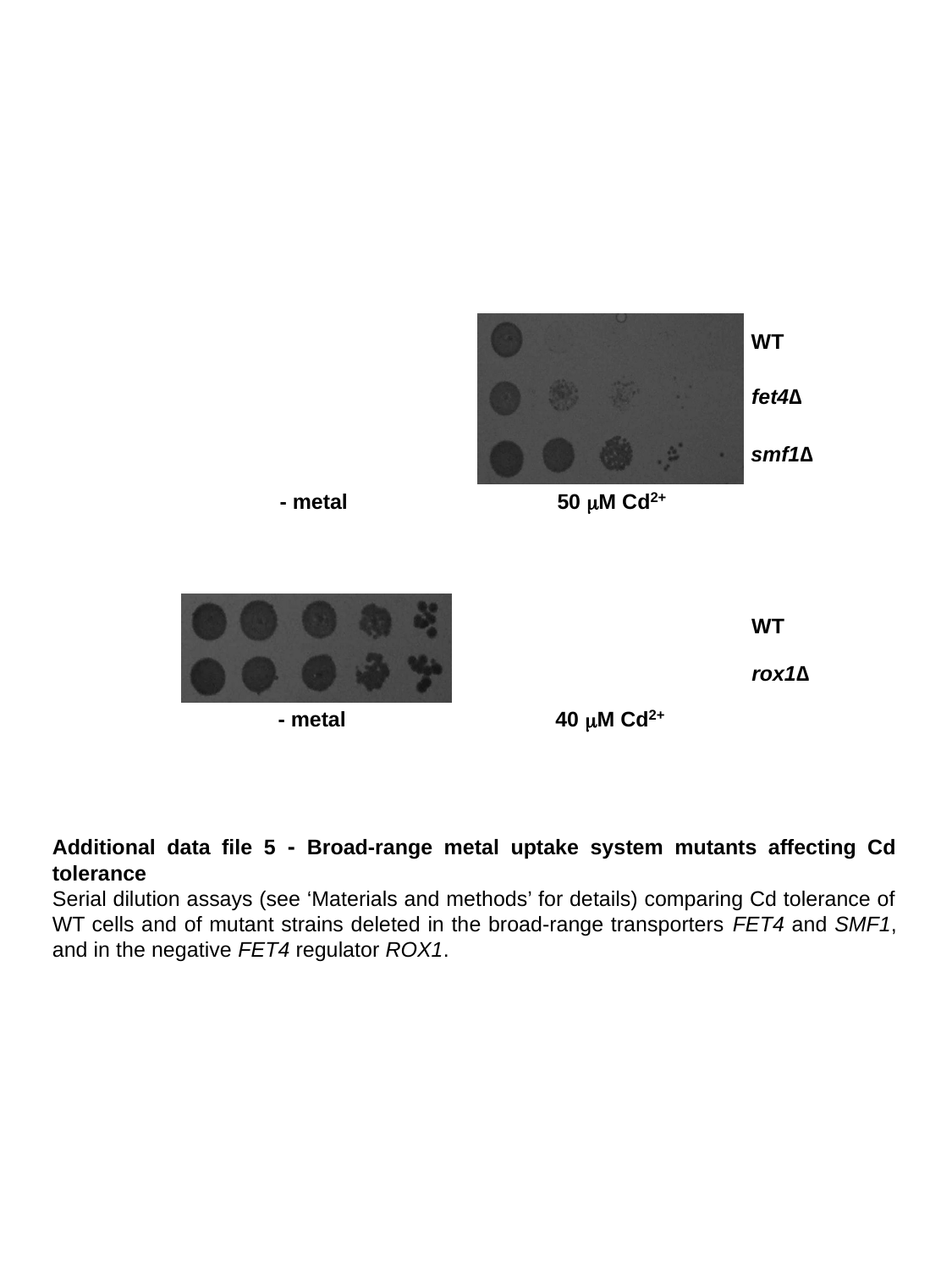

Additional data file 5 - Broad-range metal uptake system mutants affecting Cd tolerance
Serial dilution assays (see ‘Materials and methods’ for details) comparing Cd tolerance of WT cells and of mutant strains deleted in the broad-range transporters FET4 and SMF1, and in the negative FET4 regulator ROX1.
