# Supplementary material for: Membrane transporters and protein traffic networks differentially affecting metal tolerance: a genomic phenotyping study in yeast
Source: Genome Biol. 2008 Apr 7;9(4):R67. doi: 10.1186/gb-2008-9-4-r67 (PMC2643938; doi:10.1186/gb-2008-9-4-r67)
Supplement: Additional data file 6 — This figure documents the altered nickel tolerance of the fur4Δ and tna1Δ mutant strains. [file gb-2008-9-4-r67-S6.ppt]

## Slide 1
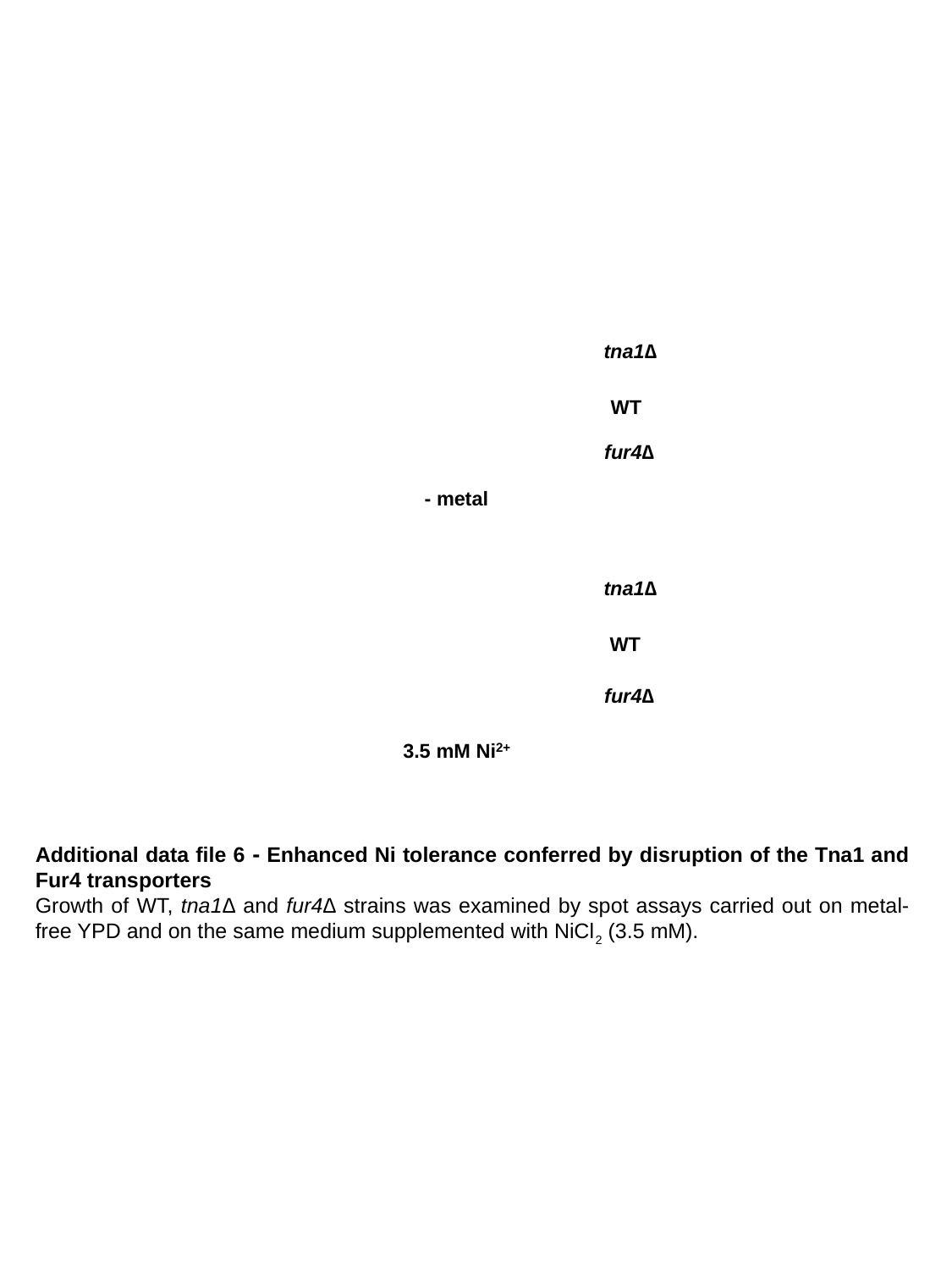

Additional data file 6 - Enhanced Ni tolerance conferred by disruption of the Tna1 and Fur4 transporters
Growth of WT, tna1∆ and fur4∆ strains was examined by spot assays carried out on metal-free YPD and on the same medium supplemented with NiCl2 (3.5 mM).
